# Effectiveness of a case-based digital learning interprofessional workshop involving undergraduates in medical technology, radiological science, and physical therapy: A pre–post intervention study

**DOI:** 10.1371/journal.pone.0270864

**Published:** 2022-07-26

**Authors:** Kazuhiro Miyata, Yuichi Aita, Syuichi Nakajima, Michiharu Sekimoto, Yukako Setaka, Yoshika Tagoya, Toshiyuki Aoyama, Takami Maeno, Masahiko Monma, Kazuhide Tomita, Haruhiko Ninomiya

**Affiliations:** 1 Department of Physical Therapy, Ibaraki Prefectural University of Health Sciences, Ami, Ibaraki, Japan; 2 Department of Medical Sciences, Faculty of Medicine, University of Tsukuba, Tsukuba, Ibaraki, Japan; 3 Department of Radiological Sciences, Ibaraki Prefectural University of Health Sciences, Ami, Ibaraki, Japan; 4 Department of Radiological Technology, Niigata University of Health and Welfare, Niigata-city, Niigata, Japan; 5 Faculty of Medicine, University of Tsukuba, Tsukuba, Ibaraki, Japan; Flinders University, AUSTRALIA

## Abstract

All healthcare professionals must understand information on a patient’s biophysical functions, and it is important to educate professionals on how to use this information in an interprofessional team for diagnosis. However, there is little interprofessional education for students of medical technology and radiological science involved in biophysical function diagnosis. In the present study, we developed a case-based interprofessional learning tool for using biophysical information for diagnosis. The study examined the effects of a collaborative exercise workshop for healthcare professional students using the tool. Participants were 234 students from three healthcare professions (medical technology, radiological science, and physical therapy). They completed the Japanese version of the Readiness for Interprofessional Learning Scale before and after the workshops. The workshops incorporated digital materials that allowed students to examine the test results of a virtual patient, answer questions, and discuss their diagnoses and prognoses. For analysis, a two-way analysis of variance was performed on the total score on the Readiness for Interprofessional Learning Scale of the three departments, and the effectiveness of the workshop for the three departments was compared. Statistical analyses showed no interaction between time and department (p = 0.283). After the workshop, students from all three departments showed significant improvements in total scores on the Readiness for Interprofessional Learning Scale *(p* < 0.01) with medium to large effect sizes (*r* = 0.33–0.52). In the comparison between departments, there was a significant difference in the awareness levels of only medical technology and radiological science students before the workshop (p = 0.015). This study conducted case-based learning workshops with students from three departments, in which a patient’s biophysical information was conveyed between occupational practices. The workshops improved the awareness of interprofessional education in students from all departments and revealed that interprofessional education is important for healthcare professions involved in biophysical function diagnosis.

## Introduction

Interprofessional education (IPE) helps students improve their collaborative practice and patient care [1]. IPE occurs “when students from two or more professions learn with, from and about each other to improve collaborative practice and the quality of care” [2]. Interprofessional frameworks, such as the World Health Organization, and their policies encourage educational institutions to incorporate IPE into their curricula [3, 4], and the evidence accumulated by empirical studies suggests that IPE exerts a beneficial impact on students [5–7]. To promote collaborative practice and patient care, it is essential to enhance training in interprofessional collaboration, starting at the pre-licensure stage.

The disciplines of medicine and nursing are most frequently involved in IPE [8]. Conversely, students dealing with information on biophysical functions, such as medical technology (MT) and radiological science (RS), were less engaged in IPE [9]. Although there are reports of IPE between radiological technologists and radiologists, their expertise is limited to IPE in very close areas [10]. All healthcare professionals must understand information on a patient’s biophysical functions, and it is important to educate professionals on how to use this information in an interprofessional team for diagnosis [11]. MT and RS can accurately capture a patient’s biophysical information, aiding comprehensive evaluation of their clinical condition and leading to appropriate treatment and care protocols. In many cases, in Japan, the same university does not have a department for MT or RS, although post-graduate education is provided for each professional. As a result, there is little interaction between professionals, and a student’s classroom knowledge and clinical practice may not be aligned.

As rehabilitation professionals, interprofessional collaboration is important for physical therapists, and they participate in many IPEs [9]. Physical therapy (PT) involves assessing the physical and motor function of a patient to plan an appropriate treatment program. Nevertheless, in actual clinical practice, prognosis prediction and program planning are carried out by determining the condition of the patient by integrating laboratory tests and diagnostic imaging findings. Therefore, PTs must be able to interpret biophysical function information (laboratory findings and imaging findings) in interprofessional collaboration.

In 2014, as part of the Problem-Solving Oriented Training Program for Advanced Medical Personnel project implemented by the Ministry of Education, Culture, Sports, Science and Technology, two universities, the University of Tsukuba and the Ibaraki Prefectural University of Health Sciences, launched the Coordinated, Continuing, Medical Staff Education Program (CoMSEP). This program provides a framework for educating undergraduate students and healthcare professionals in biophysical diagnosis support. In addition to MT and RS, which support the diagnosis of biophysical functions with imaging and other tests, PT is also included in CoMSEP. Understanding physiological function tests is important for the rehabilitation of patients with disorders, such as cardiac issues, and the exchange of knowledge between MT and PT professionals is helpful in this regard. In Japan, there are only two national-level qualifications for health technologists: medical technologists and radiological technologists. People who have acquired these qualifications can specialize further in a range of sub-fields, such as cardiovascular technologist, diagnostic medical sonographer, nuclear medicine technologist, and magnetic resonance imaging technologist, by passing the qualification exams conducted by specialist societies. Therefore, CoMSEP covers an extensive array of occupational fields. The goal of CoMSEP is to improve the quality of patient care by understanding the role of multiple professions through the interaction of previously unaffiliated medical staff.

IPE has demonstrated that smaller groups, problem-based learning, and related educational content are more effective [9, 12, 13], and patient scenarios are often used in workshops [14–16]. However, there have been no effective learning and group-work tools for biophysical diagnoses. Accordingly, the CoMSEP interprofessional workshop aimed to develop a digital learning tool in which students from the three departments (excluding medical students) work together in small groups to address clinical cases [17]. In this workshop, undergraduate students can fulfill their expert roles in a healthcare team, discuss with members from different professions how to incorporate a patient’s biodata and information into diagnosis and treatment plan, listen to one another’s professional perspectives, learn how important it is to work as a team and empower all stakeholders, and establish a care plan.

The aim of this study, thus, was to investigate whether collaborative exercise workshops using biophysical information from three departments (MT, RS, and PT) improve readiness for interprofessional learning.

## Materials and methods

### Study design

The present pre–post intervention study was a descriptive investigation designed to test the effects of the Biophysical Diagnosis Workshop on students’ attitudes toward interprofessional collaboration for patient diagnosis and care.

### Participants

A total of 234 students participated in two-day workshops conducted in the 2017 and 2019 academic years (n = 120 and n = 114, respectively). All the participants were enrolled in the third year of their undergraduate program.

### Overview of biophysical diagnosis workshop

The workshop participants were in the second half of their third undergraduate year, had received basic education in their profession, and had experienced clinical training in hospitals before the workshops. The purpose of the workshop was to demonstrate the expertise of their profession, to understand the role of other professions, and to learn the importance of interprofessional communication through discussion of cases, involving biodata and information. This workshop was compulsory and included MT students from the University of Tsukuba and RS and PT students from Ibaraki Prefectural University of Health Sciences.

The schedule for the Biophysical Diagnosis Workshop is shown in S1 Table in S1 File. During orientation, the students were advised to adopt a discussion approach that would ensure smooth interprofessional collaboration. Following icebreaker and group work, they assembled in allocated rooms for “core time” sessions. These sessions took place during both days of the workshop. Each group was presented with one clinical scenario. The clinical scenarios were based on real patient cases from the University of Tsukuba Hospital and Ibaraki Prefectural Central Hospital. After de-identification, digital material that linked these scenarios with biodata was saved to USB memory drives. In addition, image data—such as CT and MRI—were provided to the students using digital imaging and communication in a medicine viewer. The students accessed the digital material via their PCs and examined the images and diagnostic data to derive relevant diagnoses and prognoses. During these core time sessions, faculty moved between rooms to inspect the students’ progress but refrained from intervening in their discussions. If something was unclear, students were permitted to access textual materials, which the librarians and instructors from each university had prepared beforehand. For scenario tasks, they were assigned to interprofessional groups consisting of approximately 10 students, including at least three students from each department. Faculty from each department also participated as facilitators.

### Development of the interprofessional digital case-based learning tool

**Fig 1:**
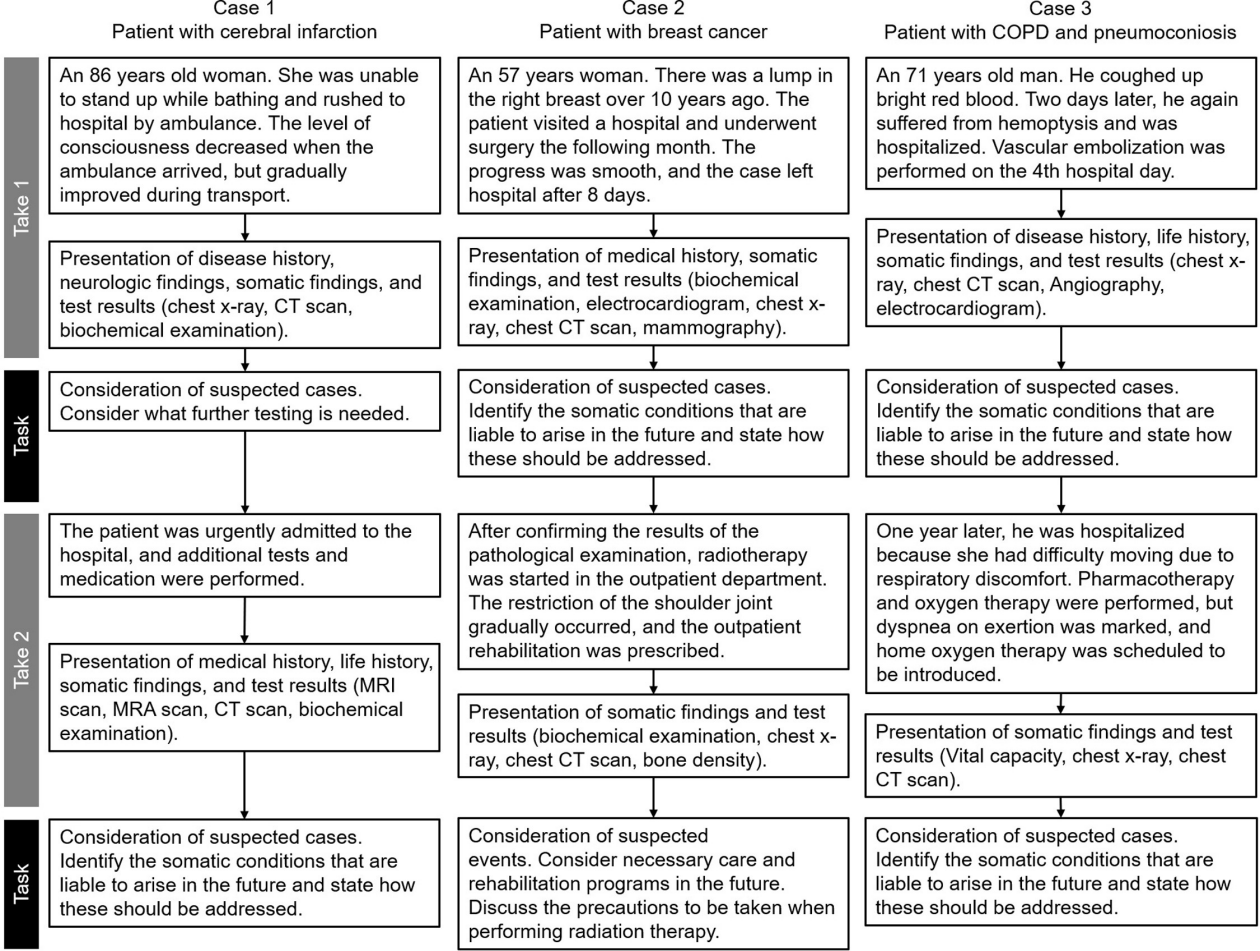
Outline of the scenario for each case.

The digital learning tool was developed for the discussion similar to case conferences in hospitals. Fig 1 shows the following clinical scenarios: 1) cerebral infarction, from diagnosis in the acute phase to rehabilitation; 2) breast cancer, postoperative pathology, and risk management of secondary complications; 3) chronic obstructive pulmonary disease and pneumoconiosis, assessment of acute phase and one year later, and subsequent pulmonary rehabilitation. The students examined virtual patients reconstructed from the digital material through tracking clinical test results over time, guided by questions. During the scenario tasks, the students had to interpret a patient’s biodata to derive a diagnosis and then determine how best to treat the patient. For this purpose, they had to confer with each other, exchange the perspectives of their respective disciplines, and finally coordinate their opinions.

### Data collection

Students completed the Japanese version of the Readiness for Interprofessional Learning Scale (RIPLS) [18, 19] at the beginning and end of the workshops, and their responses were compiled to evaluate the change in students’ attitudes toward interprofessional collaboration. The questionnaires were collected immediately after completion. The RIPLS is used to examine changes in attitudes arising from participation in learning experiences designed to improve team collaboration. The scale has been validated with students pursuing undergraduate professional education [18] and is recommended for use [20]. The items are measured on a five-point Likert scale (1 = *Strongly disagree*, 2 = *Disagree*, 3 = *Undecided*, 4 = *Agree*, and 5 = *Strongly agree*). In this study, we used the 19-item Japanese version of the RIPLS developed by Tamura et al. [19]. The scale consists of three subscales: teamwork and collaboration (13 items), IPE opportunities (2 items), and uniqueness of profession (4 items). Regarding the reliability of the RIPLS, Cronbach’s alpha coefficient is 0.83–0.89 [21], but Cronbach’s alphas for some subscales are as low as 0.60 [19]. The primary outcome was, therefore, the total RIPLS score.

### Data analyses

Data were analyzed using IBM SPSS 28 (IBM Corp., Armonk, NY) and Microsoft Excel (Microsoft Corporation, Redmond, WA) for effect size (ES) calculation. The missing values in the RIPLS responses were supplemented using a multiple imputation method. Mean scores and standard deviations were calculated for the RIPLS totals and scores on the subscales (reversed-item scores were corrected before mean score calculation). For the total RIPLS score, a two-way ANOVA was performed to compare the effects of the workshops in the three departments. If no interaction was observed, a two-tailed paired t-test was performed pre- and post-workshop, and one-way analysis of variance and post hoc comparisons (Tukey) were performed for the three departments. For the subscales, the t-test was used to compare only pre- and post-workshop. The ES was *r* for the t-test; this ES was considered small (*r* = 0.1), medium (*r* = 0.3), or large (*r* = 0.5). A *p*-value of <0.05 was considered statistically significant.

### Ethics approval

This study was approved by the Ethics Committee of the Faculty of Medicine, University of Tsukuba (#1200), and the Ethics Committee of Ibaraki Prefectural University of Health Sciences (#e127). Written informed consent was obtained from all the participants. Survey responses were kept confidential and names and other identifying information were removed for analysis.

## Results

### Participants’ characteristics and survey responses

Of the 234 students who participated in this study, 75 (32%) were MT students, 79 (34%) were RS students, and 80 (34%) were PT students. Of these students, 137 (59%) were women. All the students attended the Biophysical Diagnosis Workshop and responded to the RIPLS before and after the workshop. Seven students (2.9%) had missing data on the RIPLS.

### Differences in attitude before and after the Biophysical Diagnosis Workshop

**Fig 2:**
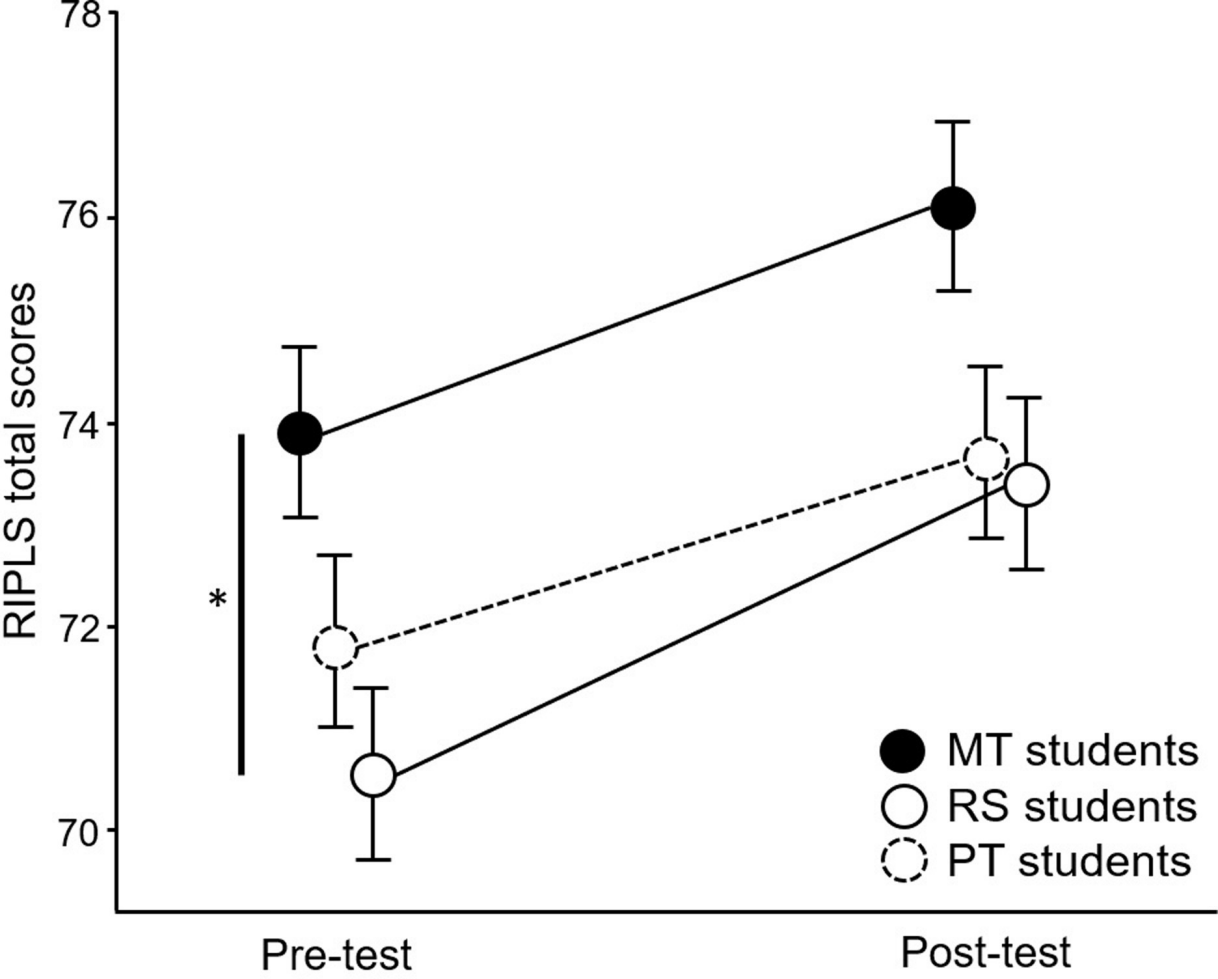
Pre and post-workshop RIPLS total scores.

Fig 2 and Table 1 show the RIPLS total scores before and after the workshop. The two-way ANOVA showed no interaction between time and department (p = 0.283). In the post-workshops, the students in all three departments showed significant improvements in the RIPLS total scores: the mean difference was 2.19 (95%CI 1.14–3.25, p < 0.001) for MT students, 2.91 (95%CI 1.80–4.02, p < 0.001) for RS students, and 1.71 (95%CI 0.67–2.76) for PT students. The ES was large in RS students (0.52), whereas it was medium in MT and PT students (0.41 and 0.33, respectively). In the comparison between departments, there was a significant difference between MT and RS students before the workshop (p = 0.015).

**Table 1:** RIPLS results for each department before and after the Biophysical Diagnosis Workshop.

	Department	Pre-test	Post-test	P-value	ES
RIPLS Total	MT	73.9 (7.6)	76.1 (6.8)	< 0.001	0.41
	RS	70.5 (7.1)	73.4 (7.6)	< 0.001	0.52
	PT	71.9 (7.4)	73.6 (7.3)	0.004	0.33
Teamwork and collaboration	MT	52.3 (5.9)	54.3 (5.3)	< 0.001	0.48
	RS	50.1 (5.4)	52.8 (5.8)	< 0.001	0.58
	PT	50.9 (5.5)	52.6 (4.9)	< 0.001	0.41
IPE opportunities	MT	7.8 (1.5)	8.2 (1.6)	0.002	0.33
	RS	7.3 (1.5)	7.6 (1.6)	0.056	0.18
	PT	7.4 (1.5)	7.6 (1.7)	0.151	0.12
Uniqueness of profession	MT	13.8 (1.3)	13.6 (2.0)	0.186	0.11
	RS	13.1 (1.7)	13.0 (1.8)	0.345	0.05
	PT	13.5 (2.0)	13.4 (2.2)	0.219	0.09

Note: All values are given as means (SD)

Abbreviations: MT, medical technology; PT, physical therapy; RIPLS, Readiness for Interprofessional Learning Scale; RS, radiological science.

The results of the RIPLS subscales are shown in Table 1. For “teamwork and collaboration,” there were significant improvements in all departments (p < 0.001), and the effects were medium to large (0.41 to 0.58). “IPE opportunities” showed significant improvement in MT alone (p = 0.002). Regarding the “uniqueness of profession” subscale, there were no significant differences before and after the workshop.

## Discussion

Educational institutions must identify effective strategies that enable students to prepare for interprofessional practice. This study developed a group-work tool for case-based learning through biophysical diagnosis. It found that students in all three departments improved their awareness of interprofessional collaboration after participating in a workshop that utilized the group-work tool. To the best of our knowledge, this study is the first to indicate that it is possible to effectively raise awareness of interprofessional collaboration among non-physician medical staff by conducting intensive case-based workshops on the biophysical diagnosis.

Our results show that RS students increased the RIPLS total score and subscale scores of “teamwork and collaboration.” A previous study [10] found that to improve the quality of radiological technologists, it was essential to improve the quality of education for radiologists, and this required a mutual exchange of information between radiologists and radiological technologists. These cases assumed that if the radiologist conveyed information to the radiological technologist on matters of clinical significance, such as imaging findings for each case, and if the radiological technologist also conveyed information to the radiologist on the patient’s condition, such as the technical content of the diagnostic imaging apparatus and test methods, patients would receive greater benefit from their tests. Gunderman and Cuskaden [10] also suggested that the workshop was an effective educational technique to increase the communication between radiologists and radiological technologists. In Japan, radiological technologists examine radiological images under the direction of a doctor, but when radiologists are not available in emergency medical situations, such as in the middle of the night, radiological technologists must help attending emergency physicians interpret the images. As shown in Fig 1, the workshop utilized educational materials comprising multiple tasks. These tasks were initiated with biochemical data obtained by MT students to ascertain a patient’s condition. RS students then considered radiological examination methods, interpreted radiological images, communicated their findings to PT students, and predicted a patient’s prognosis. For the students who had completed their clinical training, these tasks were in line with clinical practice. There was a significant difference between RS and MT in the total RIPLS score before the workshop, but there was no significant difference after the workshop. The difference in IPE education among the universities may have influenced the results.

The MT students’ total RIPLS scores before and after the workshop were higher than the scores of RS and PT students (Table 1). It has been reported that medical technologists generally work behind the scenes; thus, IPE must be designed to enable interprofessional experiences [22]. The workshop succeeded in making MT students understand more about the significance of IPE. The scores on the subscale “IPE opportunities” significantly improved after the workshop for MT students but not for RS and PT students (Table 1). Previous studies have highlighted the need for using IPE to increase the awareness of MT students (as well as of students in other majors) regarding the role of the clinical laboratory in patient care [22, 23]. Through the workshop, MT students could recognize how radiological technologists and physical therapists utilize laboratory tests performed by medical technologists.

In this study, PT students participated in the workshop as professionals who used biophysical diagnostic information; they also had significantly higher RIPLS total scores and “teamwork collaboration” subscale scores after the workshop. This also held true for MT and RS students. These results indicate that students became aware of the importance of obtaining information related to biophysical diagnosis through greater multidisciplinary collaboration with other professionals. However, apart from positive occupational identity, the effect was smaller for PT students. This may be because PT education in Japan does not include a diagnosis. In the Japanese medical system, physical therapists evaluate and intervene in PT only under the direction and diagnoses of doctors. Japanese physical therapists do not have direct access, unlike their counterparts in countries such as the United States, and do not perform triage or diagnosis. They are therefore aware of the importance of multidisciplinary collaboration associated with biophysical diagnosis. The results of this study suggest that the workshop elicited a smaller effect in PT occupations than in occupations that assist in diagnosis.

Several existing reports relate to IPE experiences involving students’ PT departments [14, 24, 25]. A systematic review that summarized the characteristics of IPE programs found that PT was the most widely adopted occupation, exceeding nursing and occupational therapy [9]. In the PT field, physical therapists must collaborate with nurses and other rehabilitation specialists. Incorporating tasks involving collaboration with MT and RS students, and having PT students evaluate images and bioclinical data may have provided participants with new perspectives and deepened their understanding of interprofessional collaboration. This task might also have equipped them with the ability to discuss therapy and rehabilitation approaches accurately after evaluation. From this perspective, the workshop was a rare and invaluable learning opportunity for PT students.

We developed a new type of case-based IPE experience, the Biophysical Diagnosis Workshop, for students seeking to become medical technicians, physical therapists, or radiologists. We also incorporated abundant biophysical information into the clinical scenarios that included tests, physiological, and imaging data from actual patients, and devised tasks to prepare plans that would be useful for rehabilitation. The goal of the Biophysical Diagnosis Workshop was to ensure that the students considered the patients’ medical and biophysical information from an interprofessional perspective, much as they would do in a clinical conference, to create a process that generated an exchange of opinions. In general, IPE learning methods include opinion exchange-, task-, observation-, simulation-, and practical-based learning, as well as lectures [26]. Interprofessional collaboration education modeled on clinical conferences has also been implemented [27]. This study employed a case-based learning style, which is a subtype of task-based learning. Case-based learning is suited to interprofessional collaboration education in undergraduate programs [27]. We added several questions to the tool as a means of guided inquiry, which is a characteristic of case-based learning [28], guiding students in how to lead discussions. This type of operating method and tool development teaches students to both provide and receive biophysical information, enabling them to learn from each other while learning about each other’s occupations.

This study had three limitations. The first was that the workshop used three different case scenarios, and we were unable to verify the effect of each scenario. In the future, it will be necessary to modify or add case scenarios to ensure that the workshop progresses more smoothly. The second limitation was that the workshops were held for third-year students with clinical practice experience because the workshop involved case-based learning, but it is unclear whether the third year was the appropriate level. Students from three academic departments across two universities participated in this workshop, which necessitated adjusting the schedule to suit the curricula of three different academic departments, but different student cohorts should also be considered. The third limitation was that RIPLS was used to evaluate readiness for interprofessional collaboration. Although the RIPLS has been validated across a wide range of contexts and languages [18, 19, 29, 30], there are conflicts in its structural validity and internal consistency [30, 31]. Therefore, in addition to the original 19 items, several modified versions have been reported [32–34]. Although there are problems with the RIPLS, there are limited tools available in the Japanese language to evaluate the educational effect on interprofessional learning. Many studies have examined the internal validity of the total RIPLS score [18, 21, 35], suggesting that the total score can currently be used as an assessment measure. Therefore, the total score was analyzed as the primary outcome. In the future, the development of an assessment scale suitable for the evaluation of the Biophysical Diagnosis Workshop will be considered.

## Conclusions

This study conducted case-based learning workshops with students from three occupations, in which a patient’s biophysical information was conveyed between occupational practices. We developed an effective digital tool for realistic patient scenarios for biophysical diagnosis. Conducting the workshops improved the awareness of IPE in students from all departments, and it revealed that IPE was also important for professions involved in biophysical diagnosis.

## Supporting information

S1 File (DOCX)
