# Supplementary material for: Effectiveness of a case-based digital learning interprofessional workshop involving undergraduates in medical technology, radiological science, and physical therapy: A pre–post intervention study
Source: PLoS One. 2022 Jul 26;17(7):e0270864. doi: 10.1371/journal.pone.0270864 (PMC9321744; doi:10.1371/journal.pone.0270864)
Supplement: S1 File — (DOCX) [file pone.0270864.s001.docx]

**Suppl. Table S1. Results of all items of RIPLS before and after the Biophysical Diagnosis Workshop in medical technology students**

| **Question items** | | **Pre-test** | **Post-test** |
| --- | --- | --- | --- |
| 1 | Learning with other students will help me become a more effective member of a health care team | 4.0 (0.6) | 4.2 (0.5) |
| 2 | Patients would ultimately benefit if health care students worked together to solve patient problems | 4.0 (0.6) | 4.2 (0.5) |
| 3 | Shared learning with other health care students will increase my ability to understand clinical problems | 4.1 (0.6) | 4.2 (0.5) |
| 4 | Learning with health care students before qualification would improve relationships after qualification | 3.9 (0.7) | 4.1 (0.6) |
| 5 | Communication skills should be learned with other health care students | 3.8 (0.9) | 4.0 (0.8) |
| 6 | Shared learning will help me to think positively about other professionals | 4.0 (0.6) | 4.2 (0.6) |
| 7 | For small group learning to work, students need to trust and respect each other | 4.2 (0.5) | 4.3 (0.5) |
| 8 | Team-working skills are essential for all health care students to learn | 4.2 (0.6) | 4.2 (0.5) |
| 9 | Shared learning will help me to understand my own limitations | 4.1 (0.7) | 4.2 (0.6) |
| 10 | I do not want to waste my time learning with other health care students | 3.9 (0.8) | 4.1 (0.8) |
| 11 | It is not necessary for undergraduate health care students to learn together | 4.0 (0.8) | 4.1 (0.9) |
| 12 | Clinical problem solving skills can only be learned with students from my own department | 3.4 (0.8) | 3.3 (1.0) |
| 13 | Shared learning with other health care students will help me to communicate better with patients and other professionals | 4.1 (0.5) | 4.2 (0.5) |
| 14 | I would welcome the opportunity to work on small-group projects with other health care students | 3.7 (0.7) | 4.1 (0.7) |
| 15 | Shared learning will help to clarify the nature of patient problems | 4.1 (0.6) | 4.2 (0.5) |
| 16 | Shared learning before qualification will help me become a better team worker | 4.0 (0.6) | 4.2 (0.5) |
| 17 | The function of nurses and therapists is mainly to provide support for doctors | 3.0 (0.9) | 2.6 (1.0) |
| 18 | I am not sure what my professional role will be | 3.7 (0.8) | 3.8 (1.1) |
| 19 | I have to acquire much more knowledge and skills than other health care students | 3.7 (0.7) | 3.8 (0.7) |

**Suppl. Table S2. Results of all items of RIPLS before and after the Biophysical Diagnosis Workshop in radiological science students**

| **Question items** | | **Pre-test** | **Post-test** |
| --- | --- | --- | --- |
| 1 | Learning with other students will help me become a more effective member of a health care team | 3.8 (0.6) | 4.1 (0.5) |
| 2 | Patients would ultimately benefit if health care students worked together to solve patient problems | 3.9 (0.5) | 4.1 (0.5) |
| 3 | Shared learning with other health care students will increase my ability to understand clinical problems | 3.8 (0.6) | 4.1 (0.6) |
| 4 | Learning with health care students before qualification would improve relationships after qualification | 3.7 (0.7) | 4.1 (0.6) |
| 5 | Communication skills should be learned with other health care students | 3.7 (0.9) | 4.1 (0.5) |
| 6 | Shared learning will help me to think positively about other professionals | 4.1 (0.6) | 4.1 (0.5) |
| 7 | For small group learning to work, students need to trust and respect each other | 4.0 (0.5) | 4.1 (0.6) |
| 8 | Team-working skills are essential for all health care students to learn | 3.9 (0.6) | 4.0 (0.6) |
| 9 | Shared learning will help me to understand my own limitations | 3.8 (0.6) | 4.1 (0.6) |
| 10 | I do not want to waste my time learning with other health care students | 3.6 (0.8) | 3.8 (0.8) |
| 11 | It is not necessary for undergraduate health care students to learn together | 3.7 (0.8) | 3.8 (0.8) |
| 12 | Clinical problem solving skills can only be learned with students from my own department | 3.2 (0.8) | 3.1 (0.9) |
| 13 | Shared learning with other health care students will help me to communicate better with patients and other professionals | 3.9 (0.5) | 4.0 (0.6) |
| 14 | I would welcome the opportunity to work on small-group projects with other health care students | 3.7 (0.7) | 3.9 (0.7) |
| 15 | Shared learning will help to clarify the nature of patient problems | 3.8 (0.6) | 4.0 (0.5) |
| 16 | Shared learning before qualification will help me become a better team worker | 3.9 (0.5) | 4.1 (0.5) |
| 17 | The function of nurses and therapists is mainly to provide support for doctors | 2.6 (0.8) | 2.4 (0.8) |
| 18 | I am not sure what my professional role will be | 3.6 (0.8) | 3.6 (0.9) |
| 19 | I have to acquire much more knowledge and skills than other health care students | 3.7 (0.8) | 3.9 (0.9) |

**Suppl. Table S3. Results of all items of RIPLS before and after the Biophysical Diagnosis Workshop in physical therapy students**

| **Question items** | | **Pre-test** | **Post-test** |
| --- | --- | --- | --- |
| 1 | Learning with other students will help me become a more effective member of a health care team | 3.9 (0.5) | 4.0 (0.5) |
| 2 | Patients would ultimately benefit if health care students worked together to solve patient problems | 3.9 (0.5) | 4.1 (0.5) |
| 3 | Shared learning with other health care students will increase my ability to understand clinical problems | 4.0 (0.6) | 4.0 (0.4) |
| 4 | Learning with health care students before qualification would improve relationships after qualification | 3.8 (0.6) | 4.0 (0.6) |
| 5 | Communication skills should be learned with other health care students | 3.9 (0.6) | 4.0 (0.5) |
| 6 | Shared learning will help me to think positively about other professionals | 3.9 (0.6) | 4.1 (0.4) |
| 7 | For small group learning to work, students need to trust and respect each other | 4.1 (0.5) | 4.2 (0.5) |
| 8 | Team-working skills are essential for all health care students to learn | 4.1 (0.6) | 4.1 (0.5) |
| 9 | Shared learning will help me to understand my own limitations | 3.9 (0.6) | 4.0 (0.5) |
| 10 | I do not want to waste my time learning with other health care students | 3.7 (0.8) | 3.8 (0.9) |
| 11 | It is not necessary for undergraduate health care students to learn together | 3.7 (0.9) | 3.8 (0.9) |
| 12 | Clinical problem solving skills can only be learned with students from my own department | 3.2 (0.9) | 3.1 (0.8) |
| 13 | Shared learning with other health care students will help me to communicate better with patients and other professionals | 3.9 (0.5) | 4.0 (0.5) |
| 14 | I would welcome the opportunity to work on small-group projects with other health care students | 3.7 (0.6) | 4.0 (0.6) |
| 15 | Shared learning will help to clarify the nature of patient problems | 3.9 (0.6) | 4.1 (0.5) |
| 16 | Shared learning before qualification will help me become a better team worker | 3.9 (0.5) | 4.1 (0.5) |
| 17 | The function of nurses and therapists is mainly to provide support for doctors | 3.1 (0.9) | 2.9 (0.9) |
| 18 | I am not sure what my professional role will be | 3.6 (0.8) | 3.5 (1.0) |
| 19 | I have to acquire much more knowledge and skills than other health care students | 3.6 (0.8) | 3.9 (0.8) |
